# Systemic Anti–PD-1 Immunotherapy Results in PD-1 Blockade on T Cells in the Cerebrospinal Fluid

**DOI:** 10.1001/jamaoncol.2020.4508

**Published:** 2020-10-08

**Authors:** Jana Portnow, Dongrui Wang, M. Suzette Blanchard, Vivi Tran, Darya Alizadeh, Renate Starr, Ramsinh Dodia, Vivian Chiu, Alfonso Brito, Julie Kilpatrick, Paige McNamara, Stephen J. Forman, Behnam Badie, Timothy W. Synold, Christine E. Brown

**Affiliations:** 1 Department of Medical Oncology and Therapeutics Research, City of Hope Beckman Research Institute and Comprehensive Cancer Center, Duarte, California; 2 Department of Hematology and Hematopoietic Cell Transplantation, T Cell Therapeutics Research Laboratories, City of Hope Beckman Research Institute and Comprehensive Cancer Center, Duarte, California; 3 Department of Computational and Quantitative Medicine, City of Hope Beckman Research Institute and Comprehensive Cancer Center, Duarte, California; 4 Department of Cancer Biology, City of Hope Beckman Research Institute and Comprehensive Cancer Center, Duarte, California; 5 Department of Clinical Research, City of Hope Beckman Research Institute and Comprehensive Cancer Center, Duarte, California; 6 Division of Neurosurgery, Department of Surgery, City of Hope Beckman Research Institute and Comprehensive Cancer Center, Duarte, California

## Abstract

**Importance:**

Little is known about the penetration and bioactivity of systemically administered programmed cell death 1 (PD-1) antibodies in the central nervous system. Such information is critical for advancing checkpoint antibody therapies for treatment of brain tumors.

**Objective:**

To evaluate pembrolizumab concentrations and PD-1 blockade on T cells in the cerebrospinal fluid (CSF) after intravenous administration.

**Design, Setting, and Participants:**

Cerebrospinal fluid and blood samples were collected from 10 adult patients with high-grade gliomas who were participating in clinical trials of intracranially administered chimeric antigen receptor (CAR) T cells and intravenous pembrolizumab at City of Hope in Duarte, California, from 2017 through 2019. Neuropharmacokinetic and immunologic correlative studies were performed on CSF and serum samples.

**Interventions or Exposures:**

Pembrolizumab, 200 mg, was given intravenously every 3 weeks with a median of 2 cycles (range, 1-8). CAR T cells were administered intracranially every 1 to 4 weeks. Cerebrospinal fluid and blood samples were collected on the day of CAR T-cell administration and then 24 hours later for a total of 100 paired samples.

**Main Outcomes and Measures:**

Pembrolizumab concentrations were measured by enzyme-linked immunosorbent assay, PD-1 blocking on T cells by flow cytometry, and results of PD-1 blockade on CAR T-cell function by in vitro tumor rechallenge assays.

**Results:**

Of the 10 patients included in this study, the mean (SD) age was 45.7 (11.0) years, and 6 (60%) were women. Steady-state pembrolizumab concentrations in the CSF were achieved by 24 hours after initial intravenous administration, with a mean CSF:serum ratio of 0.009 (95% CI, 0.004-0.014). The CSF concentrations of pembrolizumab effectively blocked PD-1 on both endogenous T cells and intracranially administered CAR T cells in the CSF, with flow cytometric detection of surface PD-1 on the T cells decreasing from a mean (SD) of 39.3% (20.2%) before pembrolizumab to a mean (SD) of 3.8% (5.8%) 24 hours after pembrolizumab infusion. Steady-state concentrations in the CSF were maintained throughout the 21-day cycle of pembrolizumab, as was the PD-1 blocking effect, evidenced by no increase in detectable surface PD-1 on T cells in the CSF during that time period. Incubation of PD-1–expressing T cells with CSF samples from patients treated with pembrolizumab also resulted in PD-1 blockade.

**Conclusions and Relevance:**

Results of this study demonstrate steady-state concentrations of pembrolizumab in CSF after intravenous administration as well as CSF concentrations that are sufficient for blocking PD-1 on endogenous and adoptively transferred T cells. This provides mechanistic insight regarding the ability of systemically administered PD-1 blocking antibodies to modulate T-cell activity in the brain.

## Key Points

**Question:**

Are systemically administered programmed cell death 1–blocking antibodies able to penetrate and maintain bioactivity in the central nervous system?

**Findings:**

In this case series study of 10 adult patients with high-grade gliomas, intravenous administration of pembrolizumab yielded cerebrospinal fluid (CSF) concentrations that were approximately 1% of that in serum but were sufficient for blocking programmed cell death 1 on T cells in the CSF.

**Meaning:**

Systemically administered immune checkpoint blockade is able to reinvigorate T cells within the CSF compartment, supporting its bioavailability for treatment of tumors in the central nervous system and its use in combination with locoregionally delivered cellular therapies.

## Teaser

This case series evaluates pembrolizumab concentrations and programmed cell death 1 blockade on T cells in cerebrospinal fluid after intravenous administration.

## Introduction

Programmed cell death 1 (PD-1) blocking antibodies are effective against many types of cancer because of their ability to reinvigorate antitumor T-cell responses. Not only do they improve survival in patients with cancer who have systemic disease, they have also shown promising activity against brain metastases from melanoma and non–small cell lung cancer.^1,2^ Responses to anti–PD-1 therapy for primary brain tumors, such as glioblastoma, have been disappointing,^3,4^ although recent small studies have suggested clinical activity in the neoadjuvant setting.^5,6^ Improving responses to immunotherapy for patients with glioblastoma or other brain tumors requires a better understanding of the neuropharmacokinetics and neuropharmacodynamics of systemically administered PD-1 antibodies.

Chimeric antigen receptor (CAR) T cells are also being investigated as a treatment for primary and metastatic brain tumors.^7,8,9,10,11^ Our author group has been studying locoregional delivery of interleukin-13 receptor α2−targeted and ERBB2-targeted CAR T cells in patients with recurrent high-grade gliomas, and we previously reported that CAR T cells delivered intraventricularly mediated complete tumor regression in a patient with multifocal glioblastoma.^8^ However, CAR T cells can be vulnerable to functional exhaustion mediated by PD-1. The addition of PD-1 blockade might enhance the efficacy of CAR T cells against brain tumors, yet it is currently unknown whether systemically administered PD-1 antibodies can achieve sufficient concentrations in the central nervous system to potentiate locoregionally delivered T-cell therapies.

## Methods

### Patients and Sample Collections

Cerebrospinal fluid (CSF) and blood samples were collected from 10 patients with high-grade gliomas (eTable 1 in the Supplement) who were participating in CAR T-cell clinical trials with cells given either intraventricularly or both intraventricularly and intracavitary (eFigure 1 in the Supplement). All patients also received pembrolizumab, 200 mg, intravenously every 21 days (eTable 2 in the Supplement). This study was conducted in accordance with the Declaration of Helsinki and approved by the City of Hope Institutional Review Board. All patients provided written informed consent. See eMethods in the Supplement for more details.

### Sample Analyses

Concentrations of pembrolizumab in serum and cell-free CSF samples were determined using a PD-1 ligand-based enzyme-linked immunosorbent assay.^12^ Immune cells in the CSF were analyzed by flow cytometry. See eMethods in the Supplement for more details, including methods for statistical analysis.

### Statistical Analysis

**Figure 1.**
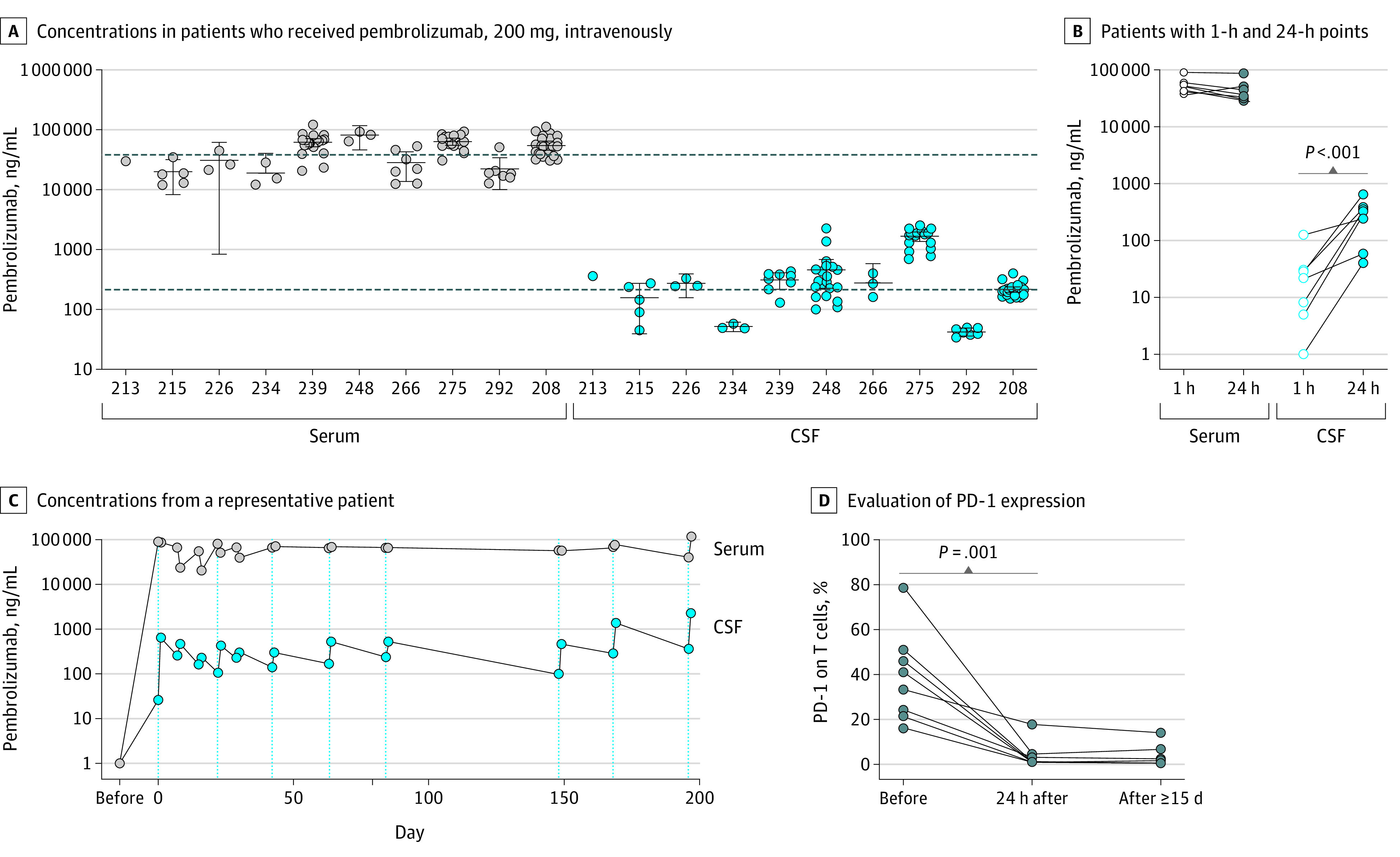
Detection of Intravenously Administered Pembrolizumab in Cerebrospinal Fluid A, Steady-state concentrations of pembrolizumab in serum and cerebrospinal fluid (CSF) from 10 patients with high-grade gliomas who received pembrolizumab, 200 mg, intravenously. All samples collected 1 day or more after the first pembrolizumab infusion are plotted. The median number of pembrolizumab cycles that patients received was 2 (range, 1-8), and the median number of paired samples collected at steady state from each patient was 6 (range, 1-25). Error bars indicate the mean 95% CIs for each patient, and the dashed line indicates the antilog of the average across all patients’ means of either serum or CSF. B, Concentrations of pembrolizumab in the serum and CSF from the 7 patients for whom 1-hour and 24-hour time points were collected after the first pembrolizumab infusion (ie, patients 213, 215, 226, 234, 239, 266, and 292). The *P* value is based on a 1-sided paired *t* test. C, Concentrations of pembrolizumab in the serum and CSF of a representative patient (patient 239) during administration of multiple cycles of pembrolizumab. Dotted lines indicate intravenous pembrolizumab infusions. D, Evaluation of programmed cell death 1 (PD-1) expression on CSF T cells. Percentages of PD-1 staining on T cells in patient CSF samples (patients 213, 215, 226, 234, 239, 268, 275, and 292) collected before (n = 8), 24 hours after the administration of intravenous pembrolizumab (n = 8), and toward the end of a pembrolizumab cycle (n = 6). The *P* value is based on a 1-sided paired *t* test.

**Figure 2.**
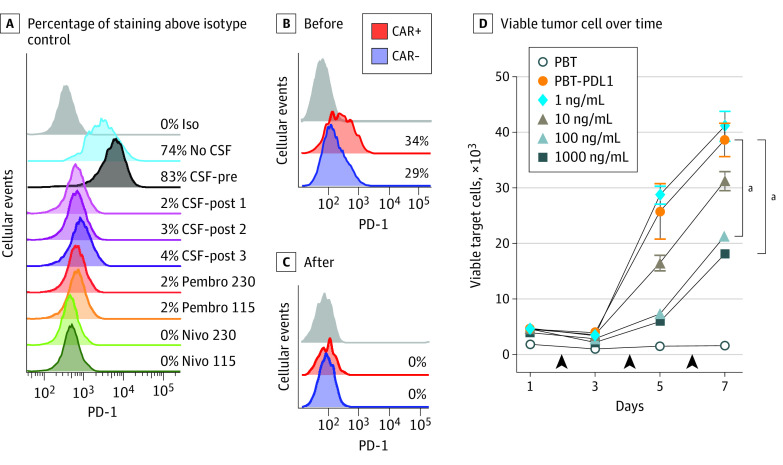
Concentrations of Pembrolizumab in Cerebrospinal Fluid Are Sufficient to Block Programmed Cell Death 1 (PD-1) on T Cells A, T cells isolated from healthy donor peripheral blood mononuclear cells were stimulated with anti–CD3/CD28 beads and incubated for 1 hour with either no cerebrospinal fluid (CSF), with CSF collected before (CSF-pre; 0 ng/mL pembrolizumab) or after intravenous pembrolizumab treatment (CSF-post 1-3; 216, 91, and 34 ng/mL pembrolizumab, respectively), or with pembrolizumab (Pembro) or nivolumab (Nivo) at either 230 ng/mL or 115 ng/mL. Cells were then stained for surface PD-1, and percentages of staining above isotype control (light gray histogram) are depicted. B-C, Flow cytometric analysis of positive chimeric antigen receptor (CAR)−gated and negative CAR−gated T cells in CSF samples of a representative patient (patient 275) collected before (B) and 21 days after the second pembrolizumab infusion (C). Percentages of CD3-gated cells staining for surface PD-1 above isotype controls (gray histograms) are depicted. D, Healthy donor-derived CAR T cells were cocultured with primary brain tumor (PBT) cells, or PBT cells overexpressing programmed cell death ligand 1 (PD-L1) with or without the indicated amount of pembrolizumab in a rechallenge assay where additional target cells (with and without pembrolizumab) were added every 48 hours (arrowheads). Viable tumor cell numbers over time are depicted. The day 7 values were compared using a 1-sided 2-sample *t* test. The *P* values were corrected to achieve a familywise error rate of .05 based on a Hochberg procedure. ^a^*P* = .03.

Statistical analyses are described in Figures 1 and 2, and in eMethods in the Supplement.

## Results

### Steady-State Concentration of Pembrolizumab in CSF

To evaluate the neuropharmacokinetics of intravenously administered pembrolizumab, we analyzed 100 pairs of CSF and serum samples. Using a PD-1 ligand enzyme-linked immunosorbent assay, we detected concentrations of pembrolizumab in serum (antilog mean, 37 905 ng/mL [95% CI, 26 462-54 297 ng/mL]; Figure 1A) that were consistent with previously reported results.^13^ Concentrations of pembrolizumab in CSF (antilog mean, 215 ng/mL [95% CI, 104-436 ng/mL]; 1.5 nM; Figure 1A) were approximately 1% of the serum (mean CSF:serum ratio, 0.009 [95% CI, 0.004-0.014]); nonetheless, CSF pembrolizumab levels were more than 2 times higher than the half maximal inhibitory concentration (0.6 nM) reported for pembrolizumab-induced PD-1 blockade.^14^

Concentrations of pembrolizumab in CSF reached steady-state levels more slowly than in the serum. For the 7 patients from whom CSF was collected within 1 hour after the start of the first intravenous infusion (antilog mean, 26 ng/mL [95% CI, 3.4-56 ng/mL]), pembrolizumab levels were significantly lower than measurements at 24 hours (antilog mean, 195 ng/mL [95% CI, 75-508 ng/mL]; one-sided paired *t* test: mean difference in log_10_ CSF, 1.16 [95% lower confidence limit, 0.73]; *P* < .001; Figure 1B). Pembrolizumab concentrations in both CSF and serum remained relatively consistent throughout each 21-day cycle (Figure 1C and eFigure 2 in the Supplement).

### Pembrolizumab Concentrations in CSF Block PD-1

We next evaluated whether the concentrations of pembrolizumab in CSF were able to block PD-1. Prior to pembrolizumab treatment, T cells in the CSF were positive for PD-1 (mean [SD], 39.3% [20.2%]). The detection of PD-1 surface expression on T cells was significantly decreased following administration of pembrolizumab (mean [SD] after 24 hours, 3.8% [5.8%]; mean difference [SE], −35.5% [7.4%]; *P* = .001; Figure 1D and eFigure 3A in the Supplement). Pembrolizumab binding to T cells was confirmed using anti–IgG 4 staining (eFigure 3B in the Supplement), demonstrating a blocking effect rather than depletion of cells expressing PD-1. Anti–IgG 4 staining was not seen on CAR T-cell products, which confirms that this antibody did not detect the IgG sequence-containing CAR (eFigure 4 in the Supplement). Furthermore, consistent with the maintained steady-state pembrolizumab concentrations (Figure 1C and eFigure 2 in the Supplement), the blocking of PD-1 on T cells in the CSF was also maintained throughout the intervals between pembrolizumab infusions (Figure 1D and eFigure 3 in the Supplement).

To confirm that CSF pembrolizumab concentrations were sufficient to block PD-1, healthy donor T cells were stimulated with CD3/CD28 Dynabeads (Thermo Fisher Scientific) to induce PD-1 expression and then incubated with CSF obtained before and after treatment with pembrolizumab. Blockade of PD-1 (Figure 2A) and detection of bound pembrolizumab (eFigure 5A in the Supplement) was only observed in CSF samples obtained after pembrolizumab administration. A similar blocking effect was seen after incubating T cells with either pembrolizumab or nivolumab, another anti–PD-1 monoclonal antibody, at concentrations similar to that measured in patient CSF (Figure 2A) and as low as 1 ng/mL (eFigure 5B in the Supplement).

We also analyzed PD-1 blockade on CAR T cells that were administered directly into the CSF. Despite initial negligible PD-1 expression on the CAR T-cell product (eFigure 6 in the Supplement), analysis of a representative CSF sample obtained prior to pembrolizumab treatment showed similar PD-1 expression on both locoregionally delivered CAR-positive T cells (administered intracavitary and/or intraventricularly) and endogenous CAR-negative T cells (Figure 2B). In CSF obtained after pembrolizumab administration, blockade of PD-1 (Figure 2C) and detection of bound pembrolizumab (eFigure 6 in the Supplement) was seen on both CAR-positive and CAR-negative T cells, demonstrating that CSF pembrolizumab concentrations were sufficient to block PD-1 on T cells.

We then evaluated the result of PD-1 blockade on CAR T-cell effector function using a patient-derived glioblastoma cell line (PBT030-2) that was lentivirally transduced to overexpress the programmed cell death ligand 1 (eFigure 7 in the Supplement). As expected, impaired CAR T cell–mediated killing efficacy was observed against glioblastoma cells overexpressing programmed cell death ligand 1 (Figure 2D). However, CAR T cell cytotoxic effects were enhanced with the addition of pembrolizumab at less than half (100 ng/mL) of the mean concentration measured in CSF (Figure 2D).

## Discussion

Recent studies have documented that intravenously administered anti–PD-1 antibodies can enhance endogenous antitumor immune responses in the brain^1,2,5,6^; however, these studies do not demonstrate whether PD-1 blockade can occur on T cells residing within the central nervous system. To our knowledge, this study is the first to report CSF concentrations of a systemically delivered PD-1 antibody and its bioactivity. Results demonstrated that PD-1 was blocked on both endogenous and intraventricularly administered CAR T cells in the CSF after intravenous administration of pembrolizumab and that CSF concentrations were sufficient to support CAR T-cell effector potency in functional assays.

Although intracerebral concentrations of PD-1 inhibitors required to produce effects on brain tumor microenvironments remain unknown, activated T cells in the CSF can traffic into the brain by extravasating from meningeal vessels and then crossing the pia mater.^15^ The finding that pembrolizumab concentrations in the CSF are sufficient to activate endogenous T cells suggests a mechanism through which systemically administered PD-1 antibodies could produce a local effect in the brain.

### Limitations

This study is limited by the small sample size. It remains possible that the observed PD-1 blockade of endogenous T cells in the CSF occurred in the systemic circulation before the cells crossed into the CSF. However, both in vitro functional assays and PD-1 T-cell blocking data establish that concentrations of pembrolizumab in the CSF are effective for blocking PD-1 on T cells.

## Conclusions

This case series study has demonstrated that CSF concentrations of systemically administered pembrolizumab can functionally block PD-1 on T cells. These results provide rationale for combining PD-1 checkpoint inhibitors with locoregionally delivered CAR T cells and other cellular therapies for the treatment of brain tumors.

## Notes

Supplement. **eMethods.** **eReferences for eMethods.** **eTable 1.** Patient summary **eTable 2.** Sampling summary for pembrolizumab measurements **eFigure 1.** Study treatment schema for the phase I CAR T cell studies (NCT02208362 and NCT03389230) **eFigure 2.** Concentrations of pembrolizumab in the serum and CSF of all other patients not depicted in Figure 1c **eFigure 3.** Loss of PD-1 staining on CSF T cells after IV pembrolizumab treatment **eFigure 4.** Anti-IgG4 reagent does not detect the IgG-containing CAR **eFigure 5.** Pembrolizumab titration **eFigure 6.** Pembrolizumab binding to T cells in the CSF **eFigure 7.** PD-L1 expression on tumor cells used in Figure 1d
