# Supplementary material for: Systemic Anti–PD-1 Immunotherapy Results in PD-1 Blockade on T Cells in the Cerebrospinal Fluid
Source: JAMA Oncol. 2020 Oct 8;6(12):1–5. doi: 10.1001/jamaoncol.2020.4508 (PMC7545351; doi:10.1001/jamaoncol.2020.4508)
Supplement: Supplement. — eMethods. eReferences for eMethods. eTable 1. Patient summary eTable 2. Sampling summary for pembrolizumab measurements eFigure 1. Study treatment schema for the phase I CAR T cell studies (NCT02208362 and NCT03389230) eFigure 2. Concentrations of pembrolizumab in the serum and CSF of all other patients not depicted in Figure 1c eFigure 3. Loss of PD-1 staining on CSF T cells after IV pembrolizumab treatment eFigure 4. Anti-IgG4 reagent does not detect the IgG-containing CAR eFigure 5. Pembrolizumab titration eFigure 6. Pembrolizumab binding to T cells in the CSF eFigure 7. PD-L1 expression on tumor cells used in Figure 1d [file jamaoncol-e204508-s001.pdf]

Supplemental Online Content

Portnow J, Wang D, Blanchard MS, et al. Systemic anti–PD-1 immunotherapy results in PD-1 blockade on T cells in the cerebrospinal fluid. *JAMA Oncol*. Published online October 8, 2020. doi:10.1001/jamaoncol.2020.4508

eMethods

eReferences for eMethods

eTable 1. Patient summary

eTable 2. Sampling summary for pembrolizumab measurements

eFigure 1. Study treatment schema for the phase I CAR T cell studies (NCT02208362 and NCT03389230)

eFigure 2. Concentrations of pembrolizumab in the serum and CSF of all other patients not depicted in Figure 1c

eFigure 3. Loss of PD-1 staining on CSF T cells after IV pembrolizumab treatment

eFigure 4. Anti-IgG4 reagent does not detect the IgG-containing CAR

eFigure 5. Pembrolizumab titration

eFigure 6. Pembrolizumab binding to T cells in the CSF

eFigure 7. PD-L1 expression on tumor cells used in Figure 1d

This supplemental material has been provided by the authors to give readers additional information about their work.

© 2020 Portnow J et al. JAMA Oncology.

Online Supplemental Material

Table of Contents:

eMethods ……………………………………………………………………….pgs. 2-7

References for eMethods…………………………………………………………...pg. 8

eTable 1…………………………………………………………..…………..pg. 9

eTable 2…………………………………………………………..……..pgs. 10-12

eFigure 1…………………………………………………………...…………pg. 13

eFigure 2…………………………………………………………...………pg. 14

eFigure 3…………………………………………………………...………pg. 15

eFigure 4…………………………………………………………...………pg. 16

eFigure 5…………………………………………………………...………pg. 17

eFigure 6…………………………………………………………...………pg. 18

eFigure 7…………………………………………………………...………pg. 19

© 2020 Portnow J et al. JAMA Oncology.

eMethods

Patient characteristics and study design

CSF and blood samples were collected from 10 patients with high-grade gliomas (HGG), nine of whom had recurrent disease and were participating in either NCT02208362 or NCT03389230 (**eTable 1** in the Supplement). The tenth patient was treated on a single subject protocol described below. The designs of the two phase I chimeric antigen receptor (CAR) T cell trials were virtually identical (**eFigure 1)** except for the length of the dose limiting toxicity (DLT) evaluation period and the CAR T cell that was administered to participants: interleukin 13 receptor alpha 2 (IL13Rα2)-targeted CAR T cells (NCT02208362) or ERBB2 (formerly HER2)-targeted CAR T cells (NCT03389230). The primary objectives of these studies were to assess the safety and feasibility of locoregional administration of CAR T cells and to determine the recommended doses for phase II testing in patients with recurrent HGG. In addition to tumor that overexpresses IL13Rα2 or ERBB2, participants must have had radiographic evidence of tumor progression more than 12 wk out from completing focal brain radiation. After manufacturing the CAR T cell product, participants underwent surgery for placement of Rickham catheter(s) for subsequent administration of the CAR T cells. During that surgery, tumor resection or biopsy was performed, as clinically indicated, to confirm tumor recurrence. Treatment with dexamethasone was allowed while on study treatment; however, participants could not take more than 6 mg/d of dexamethasone.

In both studies, the CAR T cells were initially administered weekly to patients by intraventricular (ICV), intracavitary (ICT), or both ICV and ICT delivery (depending on the treatment arm to which a patient was assigned) via Rickham reservoirs placed underneath the scalp and connected to catheters inserted into the lateral ventricle or the surgical cavity. CAR T cell dose levels ranged from 2 million to 100 million cells per catheter for each cycle. CAR T cells were administered alone on the first day of each cycle during the DLT evaluation period and afterwards for as long as the patient has available CAR T cell product as well as stable or improving disease. However, when brain imaging showed evidence of tumor progression, patients were allowed to start another therapy, such as intravenously (IV) administered pembrolizumab, while continuing with CAR T cell treatment. CSF (obtained through the Rickham catheters) and blood samples were collected prior to each administration of CAR T

© 2020 Portnow J et al. JAMA Oncology.

cells and then usually 24 h later to perform immunologic correlative studies and monitor for infection. At the cutoff date of March 31, 2019, CSF and blood samples from all study participants who had received at least one dose of pembrolizumab and from whom at least one CSF sample had been collected 24 h or more after administration of a dose of pembrolizumab were included in the analyses.

Additionally, CSF and blood samples from a patient (UPN 208) with newly diagnosed glioblastoma (GBM) treated on a single subject protocol with CAR T cells administered ICT and ICV (**eTable 1** in the Supplement) were analyzed and included in this report. This patient underwent leukapheresis prior to starting standard focal brain radiation and treatment with temozolomide (75 mg/m^2/day orally)[16] and began receiving pembrolizumab during the concurrent chemoradiation. After completing 6 wk of focal brain radiation and daily temozolomide, the patient underwent placement of two Rickham catheters (ICV and ICT) for treatment with IL13Rα2-targeted CAR T cells beginning when she resumed taking temozolomide (150-200 mg/m^2 orally on days 1-5 of a 28 d cycle) approximately 1 mo after the end of radiation. The patient also continued treatment with pembrolizumab. As with the recurrent HGG patients treated on the phase I CAR T cell studies, CSF and blood samples were collected from this patient on each day of CAR T cell administration and 24 h afterwards. All three clinical trials were approved by the City of Hope Institutional Review Board and conducted according to the Declaration of Helsinki. Each research participant gave written informed consent.

Standard adult dosing of pembrolizumab approved by the Food and Drug Administration (200 mg IV over 30 min every 3 wk) was administered to all 10 patients participating in these studies. During the time period when participants were treated with pembrolizumab, CAR T cells were allowed to be given every 1-4 wk. When administration of pembrolizumab and CAR T cells occurred on the same day, pembrolizumab was given first followed by a 10 min flush. A CSF sample was obtained approximately 1 h after the start of the pembrolizumab infusion, with a blood sample drawn in parallel, and then the CAR T cells were administered locoregionally.

Most participants were taking dexamethasone during the period of time that CSF and blood samples were collected to assess concentrations of pembrolizumab (**eTable 1**). Dexamethasone daily doses ranged from 0-6 mg, but the majority of participants took 2 mg/d or less of dexamethasone. Eight of the 10 participants underwent tumor resection rather than only a biopsy

© 2020 Portnow J et al. JAMA Oncology.

when the Rickham catheters were placed. Among the 9 patients with recurrent HGG, the median time from surgery to collection of the first set of pembrolizumab-containing CSF and blood samples was 2 mo (range 1.5 to 5 mo). With the tenth patient who had newly diagnosed glioblastoma (UPN 208), the interval of time between Rickham catheter placement and collection of those samples was shorter: 3 wk after her surgery. The median time from completing focal brain radiation and obtaining the first set of CSF and blood samples for determining the neuropharmacokinetics of pembrolizumab was 2.5 y (range 6 mo to 10 y) in the recurrent HGG patients. With UPN 208 that time interval was only 1 mo. Even though the time was relatively short between UPN 208 completing radiation and surgery and the start of collecting her CSF and blood samples, UPN 208's pembrolizumab concentrations in CSF did not substantially differ from that of other patients in the cohort (**eTable 2** and **eFigure 2**). Furthermore, while some interpatient variability existed, particularly in regard to length of time between completing focal brain radiation and starting treatment with pembrolizumab as well as co-administration of concomitant therapies (**eTable 1**), in this small cohort of patients these factors did not appear to impact the pembrolizumab neuropharmacokinetic or PD-1 blocking results.

CSF sample collection and processing

CSF and blood samples were collected from all 10 patients at the time points as indicated in **eTable 2** in the Supplement. CSF was collected through the ICV catheter, except for two patients receiving ICT-delivered CAR T cells (UPN275 and UPN292), in which CSF samples were collected through the ICT catheter, because the site communicated with the ventricular system (indicated in **eTable 2** in the Supplement). CSF samples were processed as previously described.[17] Briefly, after centrifugation, the CSF cells were resuspended in Hank's Balanced Salt Solution (HBSS) buffer (Corning CellGro) with 2% Fetal Calf Serum (Hyclone Labs) and sodium azide for immediate flow cytometric analysis; cell-free supernatants were aliquoted and stored at -80°C.

Pembrolizumab ELISA

Recombinant Human PD-1 Fc Chimera (R & D Systems, Catalog Number 1086-PD-050) was added to each well of a 96-well EIA/RIA flat-bottom plate (Corning, Catalog Number 3590) at

© 2020 Portnow J et al. JAMA Oncology.

0.5 µg/mL (100 µL/well) in coating buffer (0.1M Carbonate buffer, pH 9.6 ± 0.1). The plate was sealed and incubated in the refrigerator for 16 – 24 hours. The next day, the contents of the wells were discarded and the plate was washed twice by adding 300 uL/well of a working solution of Delfia Wash concentrate ("wash buffer", diluted 1:25 with Milli-Q water according to manufacturer's instructions, Perkin Elmer, Catalog Number 4010-0010). Each well was blocked by adding 200 µL of blocking buffer (Carbonate coating buffer with 0.5% bovine serum albumin, Sigma, Catalog Number A-9647) and incubating at room temperature for 2 ± 0.5 hours with the plate sealed. After blocking, the plates were rinsed twice with the wash buffer by adding 300 µL/well. If plates are to be used at a later time, 250 µL of assay buffer (10 mM Tris-HCl, 0.87% Sodium chloride, 0.1% Sodium azide, 0.1% Tween-20, and 0.1% Human Serum Albumin in Milli-Q water, pH 7.8 ± 0.1) was added to each well. The plate was sealed and returned to the refrigerator for storage. Plates taken out of storage were washed once with wash buffer (300 µL/well).

All samples, standards and controls were tested in duplicate. Serum samples were diluted 1:1000 using assay buffer while cerebral spinal fluid ("CSF") samples were diluted either 1:10 or 1:20 using assay buffer. Calibrators were prepared in artificial CSF or donor human serum and diluted with assay buffer to mimic the sample matrix. An 8-point standard curve was made with stock pembrolizumab starting at 500 ng/mL and diluted serially in 2-fold dilutions down to 4 ng/mL using the diluent solution specific for the sample type. Controls consisting of assay buffer only and standard diluent solution only were included. The samples, standards and controls were added to the EIA/RIA plate, which was then sealed, covered with foil and then incubated on a rocker set on low at room temperature for 1 hour. After 1 hour, the plate was washed five times with wash buffer. 100 µL of Mouse anti-Human IgG4 Secondary Antibody conjugated to HRP (diluted 0.5 µg/mL in assay buffer, Thermofisher Scientific, Catalog Number MA1-34437) was added to each well. The plate was resealed, covered with foil and then incubated on a rocker set on low at room temperature for 1 hour. At the end of the incubation, the plate was washed five times with wash buffer. For color development, 100 µL of TMB Substrate (Sigma, Catalog Number T0440) was added to each well. The plate was covered with foil and then incubated at room temperature for 5 min. Once time was up, 100 µL of Stop Reagent for TMB Substrate (Sigma, Catalog Number S5689) was added to each well. The plate was read at 620 nm on a BMG Labtech Fluostar Optima multimode plate reader within 30 min. Background correction

© 2020 Portnow J et al. JAMA Oncology.

was performed by subtracting the OD obtained from either artificial CSF diluted 1:10 in assay buffer or serum diluted 1:1000 in assay buffer. The lower limits of quantitation (LLOQ) in CSF and serum were 40 ng/mL and 4000 ng/mL, and the lower limits of detection (LLOD), defined as the average background value plus three times the standard deviation, were 25 ng/mL and 2250 ng/mL, respectively. Results were analyzed using BMG Labtech's MARS Analysis software and graphical calculations performed by using Prism Software.

Cell Lines

Human T cells were isolated from healthy donor peripheral blood and IL13Rα2-targeted CAR T cells were made as previously described,[18] with the clinical IL13Rα2-CAR T cell product (**Figure 2b**) manufactured in accordance with NCT02208362. The low passage GBM tumor sphere line PBT030-2 has been previously described.[19] PBT030-2 cells were transduced with a lentiviral construct encoding PD-L1 and a GFP reporter gene at an MOI of 3 (sequence of construct available upon request), expanded for 2-3 wk and then underwent fluorescence activated cell sorting for the GFP+ population to generate PBT-PDL1 cells. All human cells were obtained under protocols approved by the City of Hope Internal Review Board.

Flow cytometry

Cells were stained with fluorochrome-conjugated antibodies specific for human CD3, CD8, CD45, IL-13 (BD Biosciences), PD-1 (BioLegend), IgG4 (abcam), and PD-L1 (eBiosciences) with DAPI (Invitrogen) used as a viability dye. Samples were then run on a Macsquant Analyzer (Miltenyi Biotec Inc.) and analyzed using FlowJo (FlowJo, LLC) or FCS Express (De Novo Software) software.

***In vitro* PD-1 blockade assay**

Human PBMC derived T cells were stimulated with anti-CD3/CD28 dynabeads (Thermo Fisher Scientific) as previously described.[18] After 72 h incubation, the beads were magnetically removed, and T cells were incubated in media (X-VIVO (Lonza) with 10% FBS) with or without pembrolizumab or nivolumab supplementation, or in cell-free CSF supernatants, for 1 h at 37°C. T cells were harvested and stained for PD-1 and IgG4.

© 2020 Portnow J et al. JAMA Oncology.

CAR T cell cytolytic activity assay

Evaluation of CAR T cell cytolytic activity followed a previously published protocol of repetitive tumor cell challenge.[20,21] Briefly, tumor spheres were dissociated into single cells and mixed with CAR T cells (each replicate: 4,000 CAR T cells, 16,000 tumor cells). Tumor cells (32,000 per replicate) were then added into the co-culture every 48 h. Pembrolizumab (final dose indicated in **Figure 2d**) was added at the initial co-culture and at each time point of tumor cell re-challenge. T cell cytotoxicity was evaluated by flow cytometric analysis of viable tumor cells at days 1, 3, 5 and 7 after initiation of the co-culture.

Statistical analysis

Both CSF and serum data were skewed to the right leading us to transform these variables using base 10 logarithm for the calculations of the steady state values. To calculate the steady-state means, we first calculated the mean of each participant's steady-state data and then averaged those means. We then present the antilog means and 95% confidence limits.

For the comparison of 1 h and 24 h measures, both CSF and serum data were transformed using a base 10 logarithm after adding 1 to the values as one of the 1 h values was zero. A one-sided paired t-test was used to assess the change in $\log_{10}$ CSF from hour 1 to hour 24 and the change in PD-1 surface expression from pre- to post-pembrolizumab.

In the re-challenge assay, a one-sided two-sample t test was used to assess the active (PBT-PDL1) vs control (PBT) arms. P-values were corrected using a Hochberg procedure to achieve a familywise error rate of 0.05. [22] A significance level of 0.05 was used for all tests.

Each statistical analysis is described in the figure legends and was performed using Prism v6.0 (GraphPad Software) or R version 3.6.1.[23]

© 2020 Portnow J et al. JAMA Oncology.

eReferences for eMethods

16. Stupp R, Mason WP, van den Bent MJ, et al. Radiotherapy plus concomitant and adjuvant temozolomide for glioblastoma. *N Engl J Med.* 2005;352(10):987-996.

17. Brown CE, Alizadeh D, Starr R, et al. Regression of Glioblastoma after Chimeric Antigen Receptor T-Cell Therapy. *N Engl J Med.* 2016;375(26):2561-2569.

18. Brown CE, Aguilar B, Starr R, et al. Optimization of IL13Ralpha2-Targeted Chimeric Antigen Receptor T Cells for Improved Anti-tumor Efficacy against Glioblastoma. *Mol Ther.* 2018;26(1):31-44.

19. Brown CE, Starr R, Aguilar B, et al. Stem-like tumor-initiating cells isolated from IL13Ralpha2 expressing gliomas are targeted and killed by IL13-zetakine-redirected T Cells. *Clin Cancer Res.* 2012;18(8):2199-2209.

20. Wang D, Aguilar B, Starr R, et al. Glioblastoma-targeted CD4+ CAR T cells mediate superior antitumor activity. *JCI Insight.* 2018;3(10).

21. Wang D, Starr R, Alizadeh D, Yang X, Forman SJ, Brown CE. In Vitro Tumor Cell Rechallenge For Predictive Evaluation of Chimeric Antigen Receptor T Cell Antitumor Function. *J Vis Exp.* 2019(144).

22. Bretz F, Hothorn T, and Westfall P. (2011). Multiple comparisons using R. CRC Press: Boca Rotan FL

23. R core Team (2020), R: A Language and Environment for Statistical Computing. R Foundation for Statistical Computing. Vienna, Austria. www.R-project.org

© 2020 Portnow J et al. JAMA Oncology.

eTable 1. Patient summary

Unique Patient Number (UPN)	Age	Gender	Diagnosis	Route of CAR T Cell Delivery	Dexamethasone Dose per Day[a]	Other Therapies[a]
213[b]	42	Male	Recurrent GBM	ICV	2 mg	
215[b]	61	Female	Recurrent GBM	ICV	2 mg	
226[b]	32	Female	Recurrent anaplastic astrocytoma	ICV	0 mg	bevacizumab
234[b]	46	Female	Recurrent GBM	ICV	0 mg	bevacizumab
239[b]	32	Female	Recurrent HGG	ICV & ICT	0-2 mg	bevacizumab
248[b]	49	Female	Recurrent GBM	ICV	6 mg	osimertinib
266[b]	35	Male	Recurrent GBM	ICV & ICT	0-1 mg	bevacizumab
275[b]	48	Male	Recurrent GBM	ICT[e]	0-6 mg	
292[c]	64	Male	Recurrent GBM	ICT[e]	3-6 mg	bevacizumab
208[d]	48	Female	Newly diagnosed GBM	ICV & ICT	2-6 mg	temozolomide

[a] Administered concurrent with pembrolizumab and CAR T cells.
[b] These participants participated in NCT02208362.
[c] This participant participated in NCT03389230.
[d] This participant was treated on a single subject protocol, the details of which are provided in the eMethods.
[e] The ICT catheters of these two patients communicated with the lateral ventricles, so CAR T cells were actually administered ICV, and CSF samples were obtained.

© 2020 Portnow J et al. JAMA Oncology.

eTable 2. Sampling summary for pembrolizumab measurements

UPN	Day	Sample Number	Pembrolizumab Cycle (C) Day (D)	CAR T Cycle Number	Pembrolizumab (ng/mL)	
					Serum	CSF
213	-7	1		6	0.0	0.0
	-6	2			0.0	0.0
	0	3	C1 D1[c]	7	48755	29.1
	1	4	D2		30003	364.5
215	-43	1		3	0.0	0.0
	-42	2			0.0	0.0
	0	3	C1 D1[c]	4	51737	{4.1}
	1	4	D2		35980	272.2
	7	5	D8	5	18373	90.2
	8	6	D9		19498	145.4
	14	7	D15	6	12349	45.7
	15	8	D16		13834	237.3
226	-7	1		6	0.00	0.0
	-6	2			0.00	0.0
	0	3	C1 D1[c]	7	57555	125.1
	1	4	D2		45053	248.0
	7	5	D8	8	27071	328.3
	8	6	D9		21671	245.6
234	-7	1		5	0.00	0.0
	-6	2			0.00	NA
	0	3	C1 D1[c]	6	41285	{20.1}
	1	4	D2		28639	57.1
	7	5	D8	7	12071	50.6
	8	6	D9		15704	49.1
239	-7	1		5	0.00	0.0
	-6	2			0.00	0.0
	0	3	C1 D1[c]	6	91597	26.4
	1	4	D2		86336	646.8
	7	5	D8	7	66391	254.3
	8	6	D9		23694	452.2
	15	7	D16	8	56183	160.4
	16	8	D17		20825	230.2
	22	9	C2 D1	9	82720	107.5
	23	10	D2		52290	431.4
	29	11	D8	10	68293	226.9
	30	12	D9		39548	296.8
	42	13	C3 D1	11	64993	140.4
	43	14	D2		69514	303.4
	63	15	C4 D1	12	63381	166.8
	64	16	D2		69592	525.5
	84	17	C5 D1	13	66750	235.5
	85	18	D2		67184	519.6
	148	19	C6 D1	14	58239	99.8
	149	20	D2		55541	464.0
	168	21	C7 D1	15	68649	284.6
	169	22	D2		81109	1396.9
	196	23	C8 D1	16	40689	351.5
	197	24	D2		122110	2284.4

© 2020 Portnow J et al. JAMA Oncology.

UPN	Day	Sample Number	Pembrolizumab Cycle (C) Day (D)	CAR T Cycle Number	Pembrolizumab (ng/mL)	
					Serum	CSF
248[a]	-3	NA	C1 D1		NA	NA
	0	1	D4	4	96615	199.0
	1	2	D5		85116	404.5
	18	NA	C2 D1		NA	NA
	21	3	D4	5	94823	160.6
	22	4	D5		66390	268.1
266	-7	1		5	0.00	0.0
	-6	2			0.00	0.0
	0	3	C1 D1[c]	6	39938	{6.9}
	1	4	D2		32549	317.2
	7	5	D8	7	22765	370.0
	8	6	D9		20023	439.7
	14	7	D15	8	12870	223.5
	15	8	D16		12773	285.7
	21	9	C2 D1	9	53230	127.8
	22	10	D2		46008	405.9
275[a]	-85	1		3	0.00	0.0
	-83	2			0.00	0.0
	0	3	C2 D1[c]	4	94823	226.1
	1	4	D2		86807	1043.0
	7	5	D8	5	55665	681.5
	8	6	D9		62944	1320.2
	14	7	D15	6	30957	788.0
	15	8	D16		41292	1294.7
	21	9	C3 D1	7	77474	938.4
	22	10	D2		80803	1740.5
	28	11	D8	8	78571	1678.9
	29	12	D9		44366	2014.0
	35	13	D15	9	53972	1730.0
	36	14	D16		NA	1853.4
	42	15	C4 D1	10	93061	1827.2
	43	16	D2		79798	2190.5
	49	17	D8	11	57348	2243.3
	50	18	D9		58317	2172.3
	56	19	D15	12	52659	2594.0
	57	20	D16		69921	2324.7
292	-7	1		11	0.0	0.0
	-6	2			0.0	0.0
	0	3	C1 D1[c]	12	36499	{0.0}
	1	4	D2		51592	37.8
	7	5	D8	13	18957	39.0
	9	6	D10[d]		20720	50.7
	14	7	D15	14	17568	50.4
	16	8	D17[d]		18974	45.9
	21	9	D22	15	12598	32.6
	22	10	C2 D1		15548	41.6

© 2020 Portnow J et al. JAMA Oncology.

UPN	Day	Sample Number	Pembrolizumab Cycle (C) Day (D)	CAR T Cycle Number	Pembrolizumab (ng/mL)	
					Serum	CSF
208[b]	0	1	C4 D12	1	39156	308.5
	1	2	D13		88072	393.8
	8	3	C5 D1		80166	182.8
	14	4	D7	2	50434	228.3
	15	5	D8		95200	232.8
	28	6	D21	3	43589	155.0
	29	7	C6 D1		115365	165.2
	35	8	D7	4	70600	243.2
	36	9	D8		81822	206.7
	42	10	D14	5	42580	166.0
	43	11	D15		59869	174.1
	55	NA	C7 D1		NA	NA
	56	12	D2	6	43228	149.6
	57	13	D3		40712	197.3
	63	14	D9	7	34486	209.0
	64	15	D10		31505	215.6
	70	16	D16	8	31257	156.3
	71	17	D17		30363	179.8
	77	NA	C8 D1		NA	NA
	84	18	D8	9	51196	241.4
	85	19	D9		62574	233.7
	91	20	D15	10	52792	197.4
	92	21	D16		41369	206.5
	98	22	C9 D1	11	37392	175.7
	99	23	D2		70309	246.6
	112	24	D15	12	54701	314.8
	113	25	D16		39261	263.0

NA, no sample available.
Green shading, day of first pembrolizumab infusion.
Grey shading, day of other pembrolizumab infusions.
Day 0 is when the first sample was collected after pembrolizumab was administered.
Brackets indicate values extrapolated below limit of detection.
[a] Patients received a dose of pembrolizumab prior to initiating the optional CAR T cell cycles post progression.
[b] This patient was treated on a single subject protocol and the initial dose of pembrolizumab was administered 12 weeks earlier.
[c] Cycles from which data were graphed in Figure 1d.
[d] Samples that were drawn 48 h, instead of 24 h, after CAR T cell administration.

© 2020 Portnow J et al. JAMA Oncology.

eFigure 1. Study treatment schema for the phase I CAR T cell studies (NCT02208362 and NCT03389230) The study treatment schemas of NCT02208362 and NCT03389230 are virtually identical except for the length of the dose limiting toxicity (DLT) evaluation period and the CAR T cell being assessed. In both studies, CAR T cell dose levels range from 10 million to 100 million, and Rickham catheters are used to deliver the CAR T cells by intraventricular (ICV), intracavitary (ICT), or both ICT and ICV routes, depending on the treatment arm to which the participant is assigned. A cycle length is one week, with CAR T cells administered on the first day of each cycle. Patients continue with CAR T cell treatment alone for as long as brain imaging shows stable or improving disease. When patients develop tumor progression, and if they have CAR T cell doses remaining, they can start treatment with another therapy while continuing to receive CAR T cells. Nine of the 10 patients whose data are included in this Brief Report participated in one of these phase I CAR T cell studies, and at time of tumor progression they chose to be treated with pembrolizumab (200 mg IV over 30 min every 3 weeks) along with continuing locoregional delivery of CAR T cells every 1-4 weeks. Other therapies that some patients received concurrently with pembrolizumab and CAR T cells are detailed in **eTable 1**. The tenth patient (UPN 208) was treated on a single subject protocol as described in the **eMethods**. With all 10 participants, CSF and blood samples were obtained on each day of CAR T cell administration and then typically 24 h later.

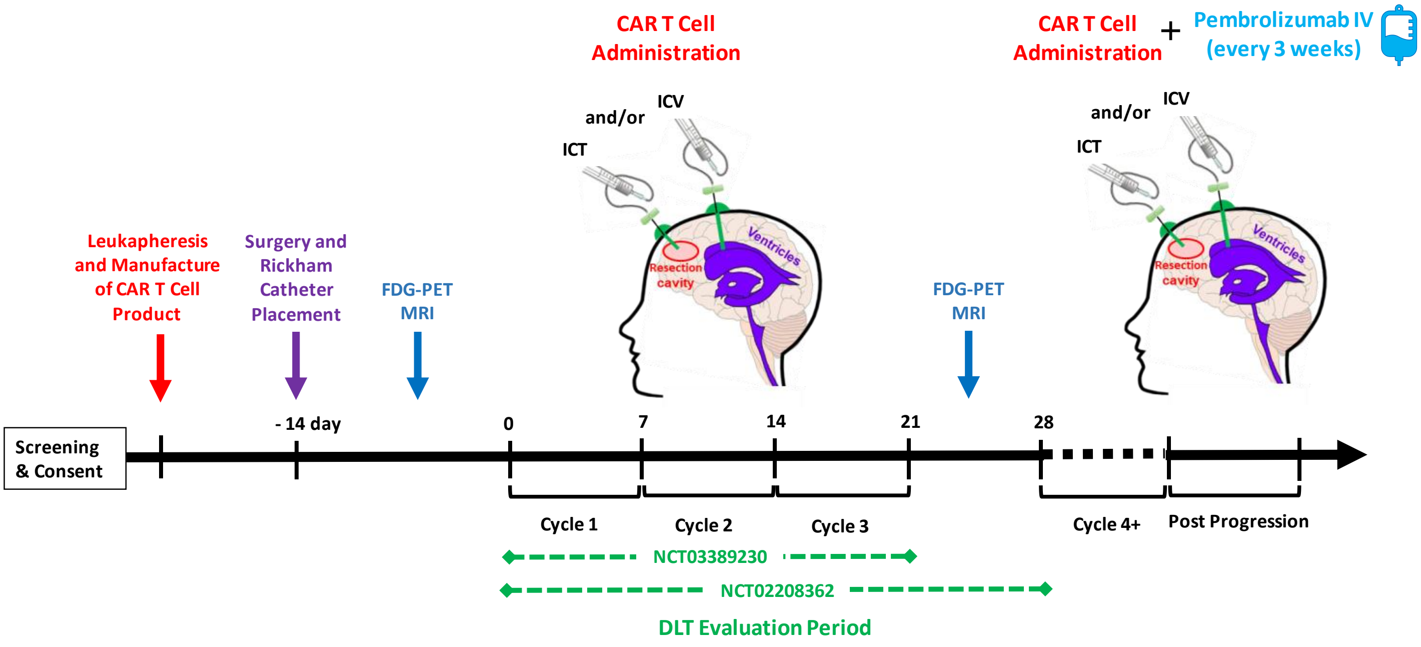

© 2020 Portnow J et al. JAMA Oncology.

eFigure 2. Concentrations of pembrolizumab in the serum and CSF of all other patients not depicted in Figure 1c. Pre-values (i.e., all values calculated as 0, reference eTable 2), are depicted as 1.0 to accommodate the log scale. Dotted lines indicate IV pembrolizumab infusions. Note patients 248, 275 and 208 had one or more pembrolizumab infusions before Day 0, making those Day 0 samples steady state samples.

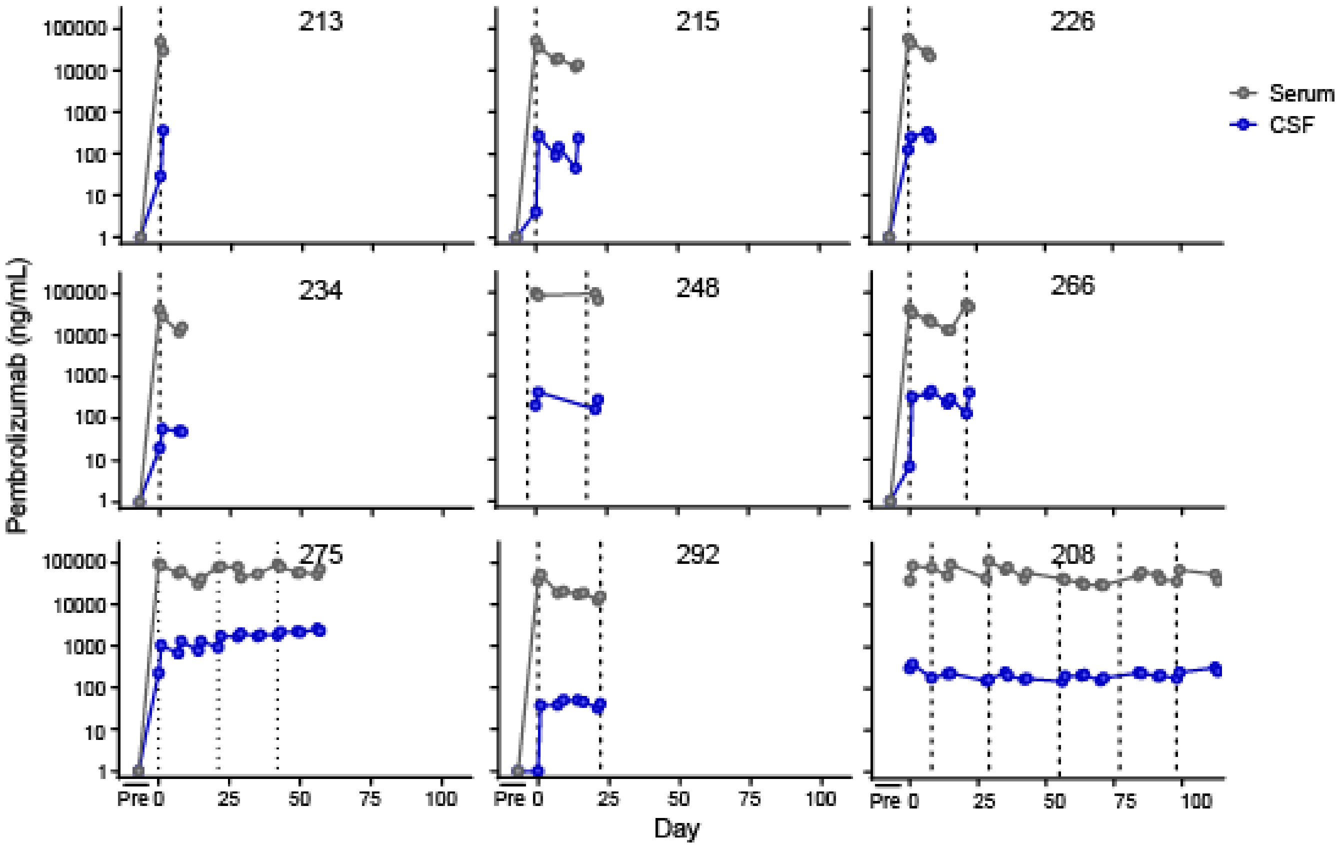

© 2020 Portnow J et al. JAMA Oncology.

eFigure 3. Loss of PD-1 staining on CSF T cells after IV pembrolizumab treatment (a) Representative flow cytometric analysis of T cells in CSF samples (UPNs 215, 239, 266, 275, 292) showing percent immunoreactivity of CD3-gated cells for CD8 vs. surface PD-1 before (Pre), 24 h after (24 h post), and toward the end of a pembrolizumab infusion cycle (Day 15+ post). (**b**) Representative flow cytometric analysis of T cells in CSF samples (UPN 275) showing percent immunoreactivity of CD3-gated cells for CD8 vs. pembrolizumab binding detected with an anti-IgG4 antibody, 24 h after (24 h post), and toward the end of a pembrolizumab infusion cycle (Day 15 post). Each histogram shows the staining intensity of cellular events as a dot plot, with quadrants drawn based on isotype/control staining.

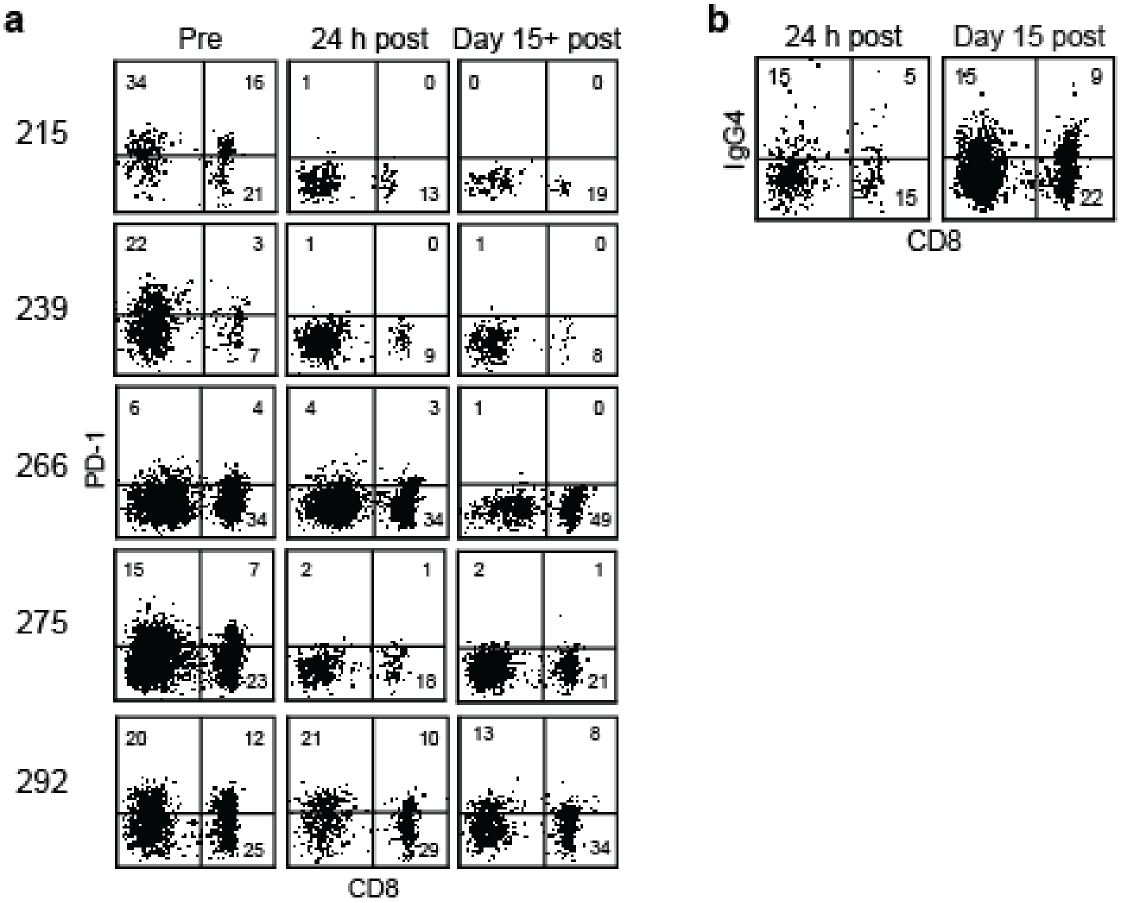

© 2020 Portnow J et al. JAMA Oncology.

eFigure 4. Anti-IgG4 reagent does not detect the IgG-containing CAR Flow cytometric analysis of three different healthy-donor derived IL13Rα2-targeted CAR T cell products using either the anti-IgG4 reagent (left) or an anti-IL13 reagent known to detect the IL13-containing CAR (right). Isotype control staining (Iso) and staining of mock-transduced T cells (Mock) were used as controls. Histogram X-axes show staining intensity while Y-axes show number of cellular events.

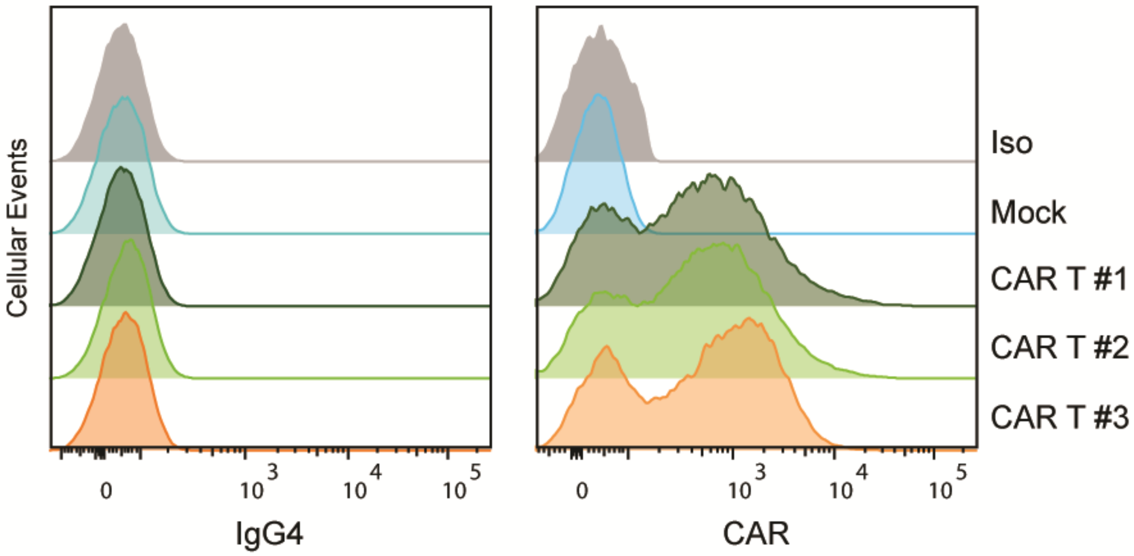

© 2020 Portnow J et al. JAMA Oncology.

eFigure 5. Pembrolizumab titration (**a**) T cells isolated from healthy donor PBMCs were stimulated with anti-CD3/CD28 beads and incubated for 1 h with either no CSF, with CSF collected before (CSF-pre; 0 ng/mL pembrolizumab) or after IV pembrolizumab treatment (CSF-post 1-3; 216, 91 and 34 ng/mL pembrolizumab, respectively), or with pembrolizumab (Pembro) or nivolumab (Nivo) at either 230 ng/mL or 115 ng/mL. Cells were then stained for pembrolizumab/nivolumab binding (IgG4) and percentages of cells staining above isotype controls (light grey histogram) are depicted. (**b**) T cells isolated from healthy donor PBMCs were stimulated with anti-CD3/CD28 beads and incubated for 1 h with pembrolizumab at either 115 ng/mL, 10 ng/mL or 1 ng/mL. Cells were then stained for surface PD-1. Percentages of cells staining for surface PD-1 above isotype controls are depicted. Histogram X-axes show staining intensity while Y-axes show number of cellular events.

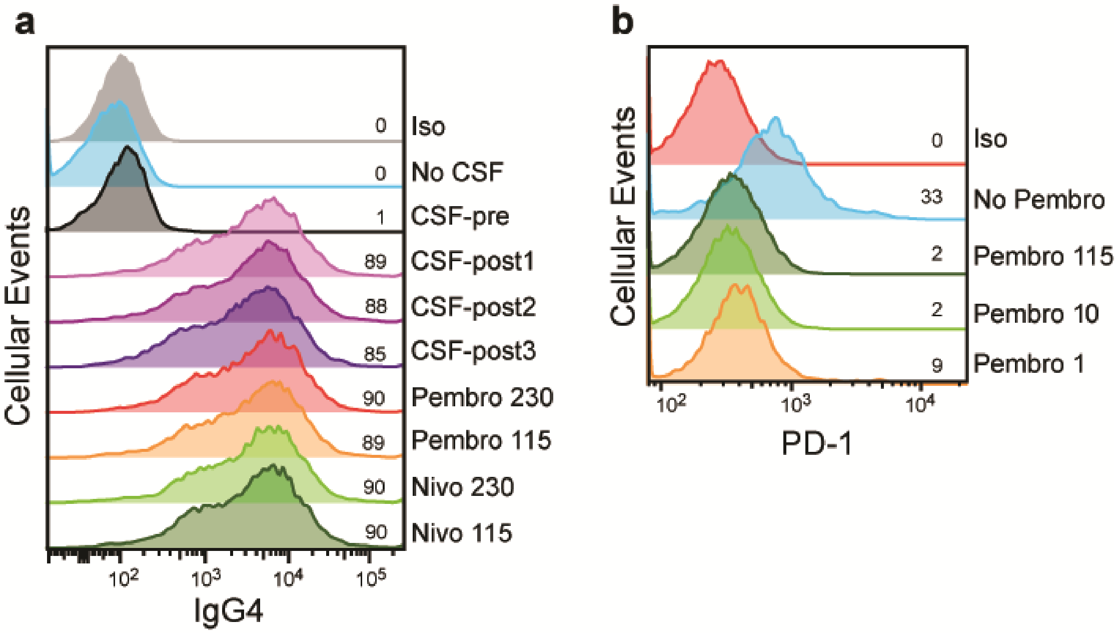

© 2020 Portnow J et al. JAMA Oncology.

eFigure 6. Pembrolizumab binding to T cells in the CSF Flow cytometric analysis of the original CAR T cell product of a representative patient (275), depicting the lack of PD-1 expression (left); and CAR-positive and CAR-negative gated T cells in CSF samples of the same representative patient (275) collected 21 days after the second pembrolizumab infusion, depicting pembrolizumab binding (right). Percentages of CD3-gated cells staining for PD-1 (left) or pembrolizumab binding (IgG4, right) above isotype controls (grey histograms) are indicated. Histogram X-axes show staining intensity while Y-axes show number of cellular events.

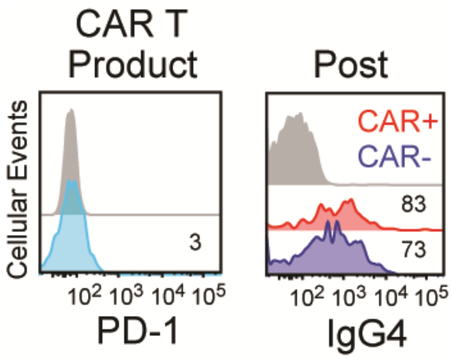

© 2020 Portnow J et al. JAMA Oncology.

eFigure 7. PD-L1 expression on tumor cells used in Figure 1d Surface staining of PD-L1 on parental PBT030-2 glioblastoma cells (PBT) or PBT lentivirally transduced to over-express PD-L1 (PBT-PDL1). Percentages of PD-L1 staining above isotype control (grey histogram) are depicted. Histogram X-axes show staining intensity while Y-axes show number of cellular events.

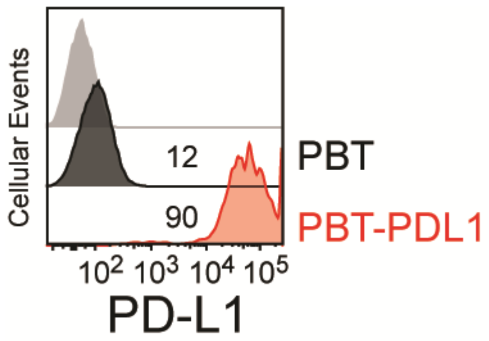

© 2020 Portnow J et al. JAMA Oncology.
